# Ancient Protostome Origin of Chemosensory Ionotropic Glutamate Receptors and the Evolution of Insect Taste and Olfaction

**DOI:** 10.1371/journal.pgen.1001064

**Published:** 2010-08-19

**Authors:** Vincent Croset, Raphael Rytz, Scott F. Cummins, Aidan Budd, David Brawand, Henrik Kaessmann, Toby J. Gibson, Richard Benton

**Affiliations:** 1 Center for Integrative Genomics, University of Lausanne, Lausanne, Switzerland; 2 School of Biological Sciences, The University of Queensland, St. Lucia, Queensland, Australia; 3 Structural and Computational Biology Unit, European Molecular Biology Laboratory, Heidelberg, Germany; Princeton University, Howard Hughes Medical Institute, United States of America

## Abstract

Ionotropic glutamate receptors (iGluRs) are a highly conserved family of ligand-gated ion channels present in animals, plants, and bacteria, which are best characterized for their roles in synaptic communication in vertebrate nervous systems. A variant subfamily of iGluRs, the Ionotropic Receptors (IRs), was recently identified as a new class of olfactory receptors in the fruit fly, *Drosophila melanogaster*, hinting at a broader function of this ion channel family in detection of environmental, as well as intercellular, chemical signals. Here, we investigate the origin and evolution of IRs by comprehensive evolutionary genomics and *in situ* expression analysis. In marked contrast to the insect-specific Odorant Receptor family, we show that IRs are expressed in olfactory organs across Protostomia—a major branch of the animal kingdom that encompasses arthropods, nematodes, and molluscs—indicating that they represent an ancestral protostome chemosensory receptor family. Two subfamilies of IRs are distinguished: conserved “antennal IRs,” which likely define the first olfactory receptor family of insects, and species-specific “divergent IRs,” which are expressed in peripheral and internal gustatory neurons, implicating this family in taste and food assessment. Comparative analysis of drosophilid IRs reveals the selective forces that have shaped the repertoires in flies with distinct chemosensory preferences. Examination of IR gene structure and genomic distribution suggests both non-allelic homologous recombination and retroposition contributed to the expansion of this multigene family. Together, these findings lay a foundation for functional analysis of these receptors in both neurobiological and evolutionary studies. Furthermore, this work identifies novel targets for manipulating chemosensory-driven behaviours of agricultural pests and disease vectors.

## Author Summary

Ionotropic glutamate receptors (iGluRs) are a family of cell surface proteins best known for their role in allowing neurons to communicate with each other in the brain. We recently discovered a variant class of iGluRs in the fruit fly (*Drosophila melanogaster*), named Ionotropic Receptors (IRs), which function as olfactory receptors in its “nose,” prompting us to ask whether iGluR/IRs might have a more general function in detection of environmental chemicals. Here, we have identified families of IRs in olfactory and taste sensory organs throughout protostomes, one of the principal branches of animal life that includes snails, worms, crustaceans, and insects. Our findings suggest that this receptor family has an evolutionary ancient function in detecting odors and tastants in the external world. By comparing the repertoires of these chemosensory IRs among both closely- and distantly-related species, we have observed dynamic patterns of expansion and divergence of these receptor families in organisms occupying very different ecological niches. Notably, many of the receptors we have identified are in insects that are of significant harm to human health, such as the malaria mosquito. These proteins represent attractive targets for novel types of insect repellents to control the host-seeking behaviors of such pest species.

## Introduction

Ionotropic glutamate receptors (iGluRs) are a conserved family of ligand-gated ion channels present in both eukaryotes and prokaryotes. By regulating cation flow across the plasma membrane in response to binding of extracellular glutamate and related ligands, iGluRs represent an important signalling mechanism by which cells modify their internal physiology in response to external chemical signals.

**Figure 1:**
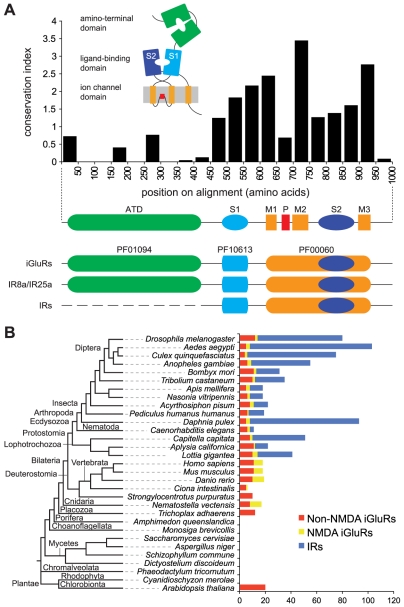
A broad phylogenetic survey of iGluR and IR genes. (A) Top: Histogram showing the mean conservation index (number of conserved physico-chemical properties) [74], [91] for 50 amino acid column-blocks of aligned *D. melanogaster* iGluRs and IRs, illustrating the higher conservation of the C-terminal region. The protein domain organisation of iGluRs/IRs is shown in cartoon form above the histogram and in linear form below it. Bottom: illustration of the three Pfam domains present in iGluRs and IRs. IR8a and IR25a contain the Pfam domain corresponding to the iGluR ATD. IR21a, IR40a, IR64a and IR93a also contain long N-termini (∼400 amino acids) but these have extremely low primary structural similarity to the ATD. All other IRs have much shorter N-terminal regions (∼200 amino acids) that lack homology to the ATD or other protein domains. (B) Histogram of the number of non-NMDA (red), NMDA (yellow) and IR (blue) sequences identified in the indicated eukaryotic species. An unscaled tree showing the phylogenetic relationships between these species is illustrated on the left.

iGluRs have originated by combination of protein domains originally encoded by distinct genes (Figure 1A) [1]–[2]. An extracellular amino-terminal domain (ATD) is involved in assembly of iGluR subunits into heteromeric complexes [3]. This precedes the ligand-binding domain (LBD), whose two half-domains (S1 and S2) form a “Venus flytrap” structure that closes around glutamate and related agonists [4]. Separating S1 and S2 in the primary structure is the ion channel pore, formed by two transmembrane segments and a re-entrant pore loop [5]. S2 is followed by a third transmembrane domain of unknown function and a cytosolic carboxy-terminal tail.

Animal iGluRs have been best characterised for their essential roles in synaptic transmission as receptors for the excitatory neurotransmitter glutamate [1], [6]. Three pharmacologically and molecularly distinct subfamilies exist, named after their main agonist: α-amino-3-hydroxy-5-methyl-4-isoxazolepropionic acid (AMPA), kainate and N-methyl-D-aspartate (NMDA). AMPA receptors mediate the vast majority of fast excitatory synaptic transmission in the vertebrate brain, while Kainate receptors have a subtler modulatory role in this process. NMDA receptors require two agonists for activation, glutamate and glycine, and function in synaptic and neuronal plasticity. Representatives of these iGluR subfamilies have been identified across vertebrates [7], as well as invertebrates, such as the fruit fly *Drosophila melanogaster*, the nematode worm *Caenorhabditis elegans* and the sea slug *Aplysia californica* [8]–[10].

While most iGluRs have exquisitely tuned synaptic functions, identification of iGluR-related genes in prokaryotic and plant genomes provided initial indication of more diverse roles for this class of ion channel. A bacterial glutamate receptor, GluR0, was first characterised in the cyanobacterium, *Synechocystis PCC6803* [11]. GluR0 conducts ions in response to binding of glutamate and other amino acids *in vitro*, suggesting a potential function in extracellular amino acid sensing *in vivo*. The flowering plant *Arabidopsis thaliana* has 20 iGluR-related genes, named GLRs [12]–[13]. Genetic analysis of one receptor, GLR3.3, has implicated it in mediating external amino acid-stimulated calcium increases in roots [14].

We recently described a family of iGluR-related proteins in *D. melanogaster*, named the Ionotropic Receptors (IRs) [15]. Several lines of evidence demonstrated that the IRs define a new family of olfactory receptors. First, the IR LBDs are highly divergent and lack one or more residues that directly contact the glutamate ligand in iGluRs. Second, several IRs are expressed in sensory neurons in the principal *D. melanogaster* olfactory organ, the antenna, that do not express members of the other *D. melanogaster* chemosensory receptor families, the Odorant Receptors (ORs) and Gustatory Receptors (GRs) [16]. Third, IR proteins localise to the ciliated endings of these sensory neurons and not to synapses [15]. Finally, mis-expression of an IR in an ectopic neuron is sufficient to confer novel odour-evoked neuronal responses, providing direct genetic evidence for a role in odour sensing [15].

The identification of the IRs as a novel family of olfactory receptors in *D. melanogaster* provides a potential link between the well-characterised signalling activity of iGluRs in glutamate neurotransmitter-evoked neuronal depolarisation and a potentially more ancient function of this family in environmental chemosensation. In this work, we have combined comparative genomics, molecular evolutionary analysis and expression studies to examine the evolution of the IRs. Four principal issues are addressed: first, when did olfactory IRs first appear? Are they a recent acquisition as environmental chemosensors in *D. melanogaster*, or do they have earlier origins in insect or deeper animal lineages? Second, what is the most recent common ancestor of IR genes? Do they derive from AMPA, Kainate or NMDA receptors, or do they represent a distinct subfamily that evolved from the ancestral animal iGluR? Third, what mechanisms underlie the expansion and diversification of this multigene family? Finally, do IRs function only as olfactory receptors or are they also involved in other sensory modalities? Through answers to these questions, we sought insights into IR evolution in the context of the origins of iGluRs, the appearance and evolution of other chemosensory receptor repertoires and the changing selective pressures during animal diversification and exploitation of new ecological niches.

## Results

### A broad phylogenetic survey of iGluR and IR genes

iGluRs and IRs are characterised by the presence of a conserved ligand-gated ion channel domain (the combined Pfam domains PF10613 and PF00060 [17]) (Figure 1A). All iGluRs additionally contain an ATD (Pfam domain PF01094), which is discernible, but more divergent, in only two *D. melanogaster* IRs, IR8a and IR25a. Most IRs have only relatively short N-terminal regions preceding the LBD S1 domain (Figure 1A). To identify novel iGluR/IR-related genes, we therefore constructed a Hidden Markov Model (HMM) from an alignment of the conserved iGluR/IR C-terminal region, which is specific to this protein family. In combination with exhaustive BLAST searches, we used this HMM to screen raw genomic sequences and available annotated protein databases of 32 diverse eukaryotic species and 971 prokaryotic genomes (see Materials and Methods and Table S2 in Supporting Information). These screens identified all previously described eukaryotic iGluRs and all *D. melanogaster* IRs, as well as 23 prokaryotic iGluRs. Novel sequences were manually reannotated and classified by sequence similarity, phylogenetic analysis and domain structure as either non-NMDA (i.e. AMPA and Kainate) or NMDA subfamily iGluRs, or IRs (Figure 1B, Table S3, and Datasets S1 and S2). Like *D. melanogaster* IRs, newly annotated IRs have divergent LBDs that lack some or all known glutamate-interacting residues, supporting their distinct classification from iGluRs.

iGluRs are widespread in eukaryotes, present in all analysed Metazoa (except the sponge, *Amphimedon queenslandica* [18]) and Plantae, but absent in unicellular eukaryotes (Figure 1B, Table S3, and Datasets S1 and S2). Analysis of iGluR subfamilies on the eukaryotic phylogeny suggests that NMDA receptors may have appeared after non-NMDA receptors, as we identified them in Eumetazoa but not in the placozoan *Trichoplax adhaerens*. Further support for this conclusion will require additional genome sequences. One member of the Eumetazoa, the sea urchin *Strongylocentrotus purpuratus*, may have secondarily lost NMDA receptors. Different species contain distinct numbers of each iGluR subfamily: vertebrates, for example, have more NMDA receptor subunits than invertebrates.

Notably, IRs were identified throughout Protostomia, encompassing both Ecdysozoa (e.g. nematodes and arthropods) and Lophotrochozoa (e.g. molluscs and annelids) (Figure 1B, Table S3, and Datasets S1 and S2). There is substantial variation in the size of the IR repertoire, from three in *C. elegans* to eighty-five in the crustacean *Daphnia pulex*. Amongst insects, Diptera (i.e. flies and mosquitoes) generally had a larger number of IRs than other species. We did not identify IRs in Deuterostomia, Cnidaria or Placozoa.

### Evolutionary conservation and expression of antennal IRs

**Figure 2:**
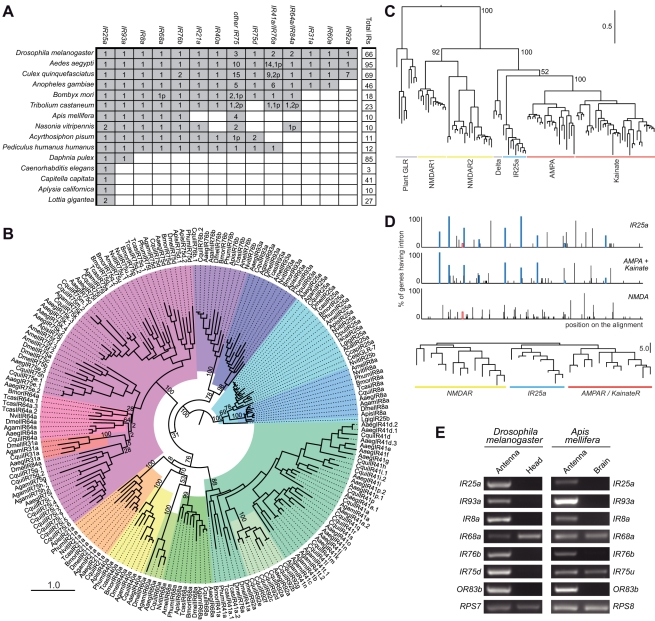
Evolutionary origins and conservation of antennal IRs. (A) Table of antennal IR orthologous groups in the indicated protostome species. A shaded square signifies the presence of at least one representative gene in a species. Figures within a square indicate the existence of multiple functional copies; the “p” suffix indicates the number of pseudogene copies. We considered as pseudogenes only those with frameshift or internal stop codons inside conserved domains due to the difficulty in accurately annotating the termini of these sequences. (B) Phylogenetic relationships of the genes shown in (A). Each colour represents a group of orthologous sequences. The sequences were aligned with PROBCONS and the tree was built with RAxML under the WAG model of substitution with 1000 bootstrap replicates. The scale bar represents the expected number of substitutions per site. (C) Phylogenetic relationships between iGluRs and all IR25a orthologues, excluding low quality or short gene annotations. The tree was built with RAxML under the WAG model of substitution, with 1000 bootstrap replicates. Bootstrap values for selected branches are shown as percentages. The scale bar represents the expected number of substitutions per site. (D) *Top:* Map of intron positions in an alignment of eight *IR25a* orthologues, 12 AMPA/Kainate receptors and 13 NMDA receptors (see Dataset S3 for alignment file). Coloured boxes illustrate introns whose positions and phases are conserved in at least one member of two different subgroups. Empty coloured boxes indicate introns conserved in position, but not in phase. *Bottom:* Phylogram based on the position of introns in the same subset of sequences as above. The scale bar represents the number of non-conserved intron positions. (E) RT-PCR analysis of antennal IR gene expression of orthologous genes (except *DmelIR75a* and *AmelIR75u*, which are paralogous genes) in *D. melanogaster* and *A. mellifera* tissues. Control RT-PCR products for comparative analysis of gene expression correspond to the ribosomal genes *RPS7* (*D. melanogaster*) and *RPS8* (*A. mellifera*). All RT-PCR products were sequenced to confirm their identity.

To explore the evolutionary origin of the IRs, we examined phylogenetic relationships of the identified protostome IRs. Reciprocal best-hit analysis using *D. melanogaster* sequences as queries revealed that a subset of this species' IRs was conserved in several distant lineages, allowing us to define putative orthologous groups. These include one group containing representatives of all protostome species (IR25a), one represented by all arthropods (IR93a), nine by most or all insects, and three by dipteran insects (Figure 2A and 2B). For most orthologous groups, a single gene for each species was identified. In a few cases, for example the IR75 group, certain species were represented by several closely related in-paralogues, some of which appeared to be pseudogenes (Figure 2A and 2B, Table S3, and Datasets S1 and S2).

Consistent with its conservation in Protostomia, IR25a is the IR with the most similar primary sequence to iGluRs, suggesting that it is the IR gene most similar to the ancestral IR. Analysis of the phylogenetic relationship of IR25a and eukaryotic iGluRs locates it clearly together with the animal iGluR family, in the non-NMDA receptor clade (Figure 2C). To substantiate this conclusion, we asked whether the *IR25a* gene structure resembles more closely that of NMDA or non-NMDA receptors. Intron positions and numbers are extremely variable across *IR25a* orthologues, with multiple cases of intron loss, gain and putative intron sliding events by a few nucleotides (Figure 2D). Nevertheless, we identified eight intron positions that are conserved between at least subsets of *IR25a* orthologues and *D. melanogaster* non-NMDA receptor genes, some of which may represent intron positions present in a common ancestral gene. By contrast, only a single intron that was conserved in position (but not in phase) was identified between *DmelIR25a* (but not other *IR25a* orthologues) and *DmelNMDAR1* (Figure 2D). A phylogram of intron positions in *IR25a*, non-NMDA and NMDA sequences reveals greater similarity of *IR25a* intron positions to those of non-NMDA receptors than NMDA receptors (Figure 2D). Together, these observations support a model in which *IR25a* evolved from a bilaterian non-NMDA receptor gene.

The conserved *D. melanogaster* IRs encompass the entire subset of its IR repertoire that is expressed in the antenna [15]. Moreover, evidence for antennal expression of the three additional genes, *DmelIR41a*, *DmelIR60a* and *DmelIR68a*, has been obtained by reverse transcription (RT)-PCR analysis, although we have not yet been able to corroborate this by RNA *in situ* hybridisation (data not shown). These combined phylogenetic and expression properties led us to designate this subfamily of receptors the “antennal IRs”.

We examined whether antennal expression of this subfamily of IRs is conserved outside *D. melanogaster* by performing a series of RT-PCR experiments on the honey bee, *Apis mellifera*, for all six putative antennal IR orthologues: *IR8a*, *IR25a*, *IR68a*, *IR75u*, *IR76b* and *IR93a* (see Materials and Methods for the nomenclature of newly-identified IRs). As in *D. melanogaster*, we could reproducibly amplify all of these bee genes from antennal RNA preparations but not in control brain RNA, except for *AmelIR68a* and *AmelIR75u*, which are also detected in the brain (Figure 2E). Thus, antennal expression of this subgroup of IRs is conserved across the 350 million years separating dipteran and hymenopteran insect orders [19], and therefore potentially in all insects.

### Conserved IR chemosensory expression in Protostomia

**Figure 3:**
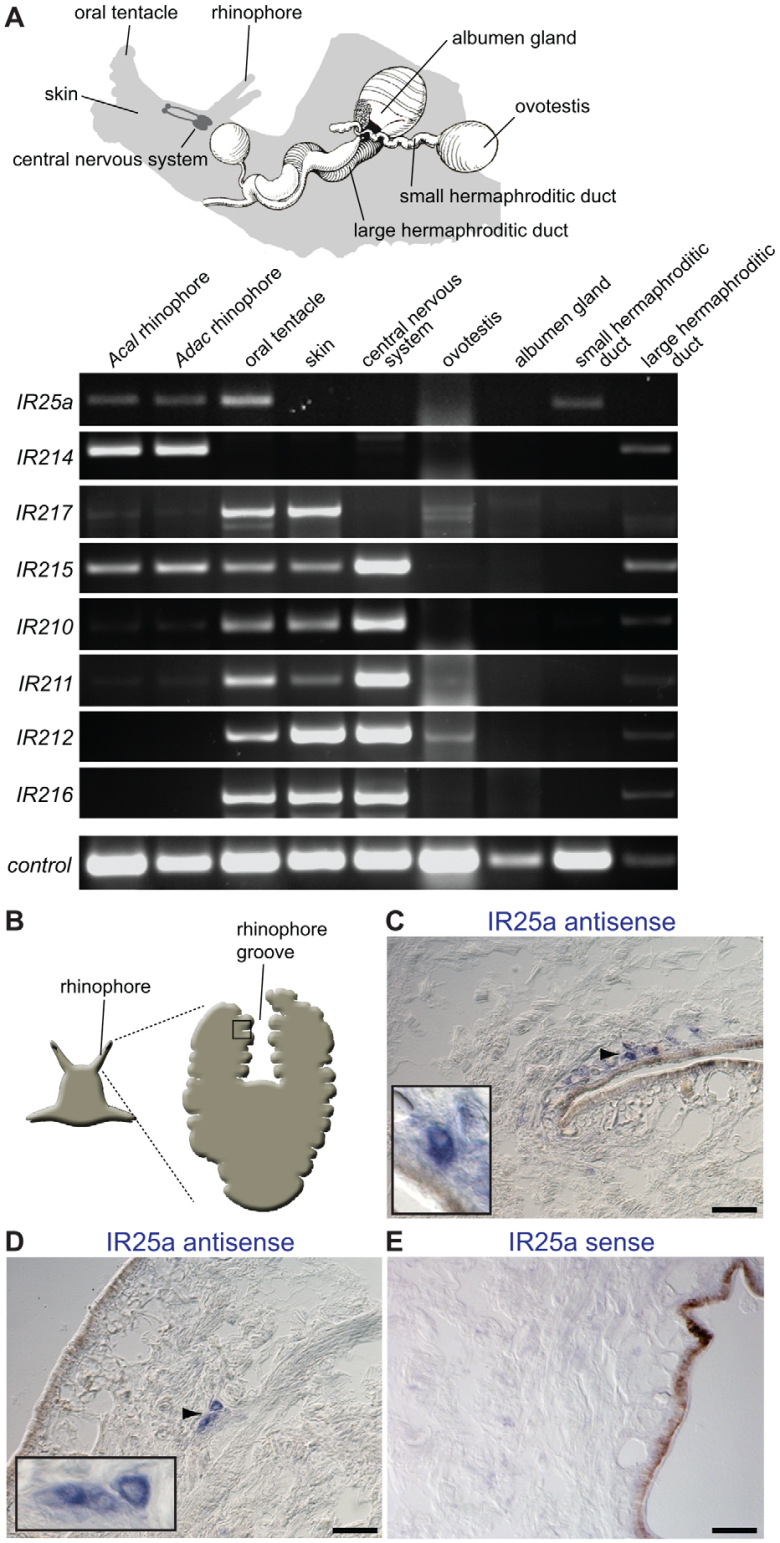
Olfactory expression of IRs in *Aplysia* molluscs. (A) *Top:* Schematic representation of *Aplysia*, illustrating the location of selected sensory, neuronal and reproductive tissues used for RNA isolation and RT-PCR (adapted from [21]). The central nervous system samples comprised pooled cerebral, pleural, buccal, pedal and abdominal ganglia. The skin samples were taken from the side of the head. *Bottom:* RT-PCR analysis of *Aplysia* IR gene expression from the indicated species and tissues. Only rhinophores from *A. californica (Acal)* were tested due to limited availability of animals, while rhinophore and other tissues were examined for the closely related species *A. dactylomela* (*Adac*) [92]. Nucleotide sequence identity of IR orthologues between these species is >85%. Control RT-PCR corresponds to β-actin. (B) Schematic of *Aplysia* rhinophore showing the approximate location of the field of views of the rhinophore groove olfactory tissue in (C–E). (C,D) RNA *in situ* hybridisation on *A. dactylomela* rhinophore sections using a digoxigenin-labelled antisense RNA probe for *AdacIR25a*. Micrographs reveal *IR25a* expression (blue) in small clusters of cells of a characteristic neuronal morphology close to the sensory epithelial surface. Higher magnifications of specific cellular staining (arrowhead) are shown in the insets. The scale bars represent 100 µm. (E) Control RNA *in situ* hybridisation on an *A. dactylomela* rhinophore section with a digoxigenin-labelled sense riboprobe for *AdacIR25a*. No signal is apparent. The scale bar represents 100 µm.

To investigate whether IRs are likely to have an olfactory function beyond insects, we examined expression of the IR repertoire from a representative of a distantly related protostome lineage, *Aplysia* molluscs, whose last common ancestor with *D. melanogaster* probably existed 550–850 million years ago [20]. We first used RT-PCR to analyse the expression of the ten *Aplysia* IR genes in a variety of sensory, nervous and reproductive tissues (Figure 3A). Notably, the *Aplysia IR25a* orthologue is predominantly expressed in the olfactory organs, the rhinophore and oral tentacle [21]. Two other *Aplysia-*specific IR genes, *IR214* and *IR217*, are expressed in the rhinophore and oral tentacle, respectively, and not detected in other tissues, except for the large hermaphroditic duct (*IR214*) and skin (*IR217*). Five additional IRs are also expressed in the oral tentacle, but displayed broader tissue expression in skin and the central nervous system; both of these tissues are likely to contain other types of chemosensory cells [22]–[23]. Expression of two IR genes, *IR209* and *IR213*, was not detected in this analysis (data not shown).

To further characterise *Aplysia IR25a*, we analysed its spatial expression in the mature *A. dactylomela* rhinophore by RNA *in situ* hybridisation. An antisense probe for *AdacIR25a* labels a small number of cells in rhinophore cryosections. Their size and morphology is typical of neurons, although we lack an unambiguous neuronal marker to confirm this identification (Figure 3B–3D). These cells are found either singly or in small clusters adjacent or close to the sensory epithelial surface in the rhinophore groove, in a similar position to cells expressing other types of chemosensory receptors [21]. A control sense riboprobe showed no specific staining (Figure 3E). Together, these results are consistent with at least some of these molluscan IRs having a chemosensory function.

The expression of putative IR25a orthologues has previously been reported in two other Protostomia. An *IR25a*-related gene from the American lobster, *Homarus americanus*, named OET-07, is specifically expressed in mature olfactory sensory neurons [24]–[25]. In *C. elegans*, a promoter reporter of the *IR25a* orthologue, *GLR-7*, revealed expression in a number of pharyngeal neurons [9], which might have a role in food sensing [26]. While both crustacean and nematode genes were classified in these studies as iGluRs, there is no evidence that they act as canonical glutamate receptors, and we suggest that they fulfil instead a chemosensory function.

### Species-specificity of divergent IRs

**Figure 4:**
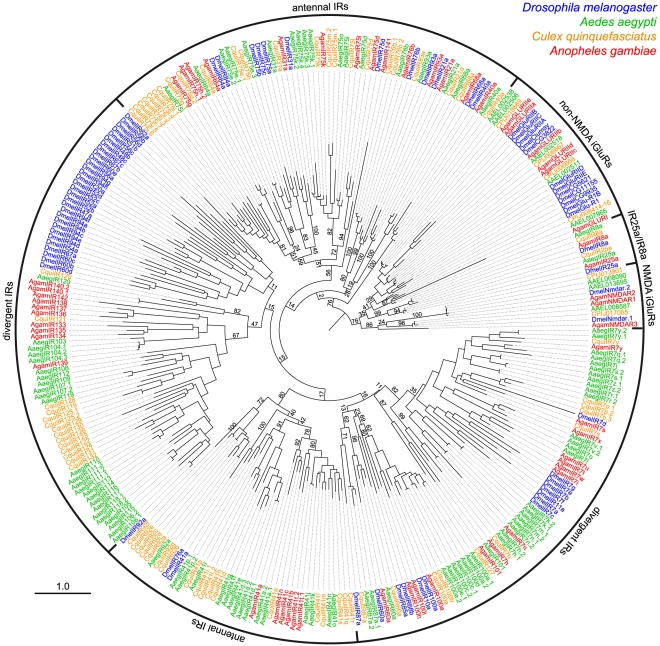
Species-specificity of divergent IR repertoires. Phylogenetic tree of all iGluRs and IRs from *D. melanogaster* (blue), *A. aegypti* (green), *C. quinquefasciatus* (orange) and *A. gambiae* (red). Sequences were aligned with PROBCONS and the tree was built with RAxML under the WAG model of substitution, with 500 bootstrap replicates. The scale bar represents the expected number of substitutions per site. Note that due to the high divergence and number of sequences analysed, bootstrap values in several of the most internal nodes are extremely low and the position of certain large clades of IR genes on the tree are distinct from trees in other figures.

The antennal IR subfamily accounts for only a small fraction of the IR repertoire in most analysed insects and only 1–2 genes in other Protostomia. The remaining majority of IR sequences are - amongst the genomes currently available - largely species-specific, with low amino acid sequence identity (as little as 8.5%) with other IR genes in either the same or different species. We refer to this group of genes here as the “divergent IRs”. Dipteran insects have particularly large expansions of divergent IRs (Figure 1B). Phylogenetic analysis revealed no obvious orthologous relationships of these genes either between *D. melanogaster* and mosquitoes or amongst the three mosquito species (*Aedes aegypti*, *Culex quinquefasciatus* and *Anopheles gambiae*) (Figure 4). Instead, this subfamily of IRs displays a number of species-specific clades, perhaps reflective of the distinct ecological niches of these insects.

### Divergent IRs as candidate gustatory receptors in adult and larval *D. melanogaster*

By contrast to antennal IRs, divergent IR expression has not been detected in *D. melanogaster* olfactory organs [15], leading us to test whether these genes are expressed in other types of chemosensory tissue. As endogenous transcripts of non-olfactory chemosensory genes, such as GRs, are difficult to detect [27]–[28], we employed a sensitive transgenic approach to investigate divergent IR expression. We transformed flies with constructs containing putative promoter regions for these genes upstream of the yeast transcription factor GAL4 and used these “driver” transgenes to induce expression of a GAL4-responsive *UAS-mCD8:GFP* fluorescent reporter [29]. We sampled divergent IRs from several distinct clades, including *IR7a*, *IR11a*, *IR52b*, *IR56a* and *IR100a* (Figure 4). All IR promoter-GAL4 constructs were inserted in the same genomic location using the phiC31 integrase system [30], eliminating transgene-specific position effects on expression resulting from their site of integration.

**Figure 5:**
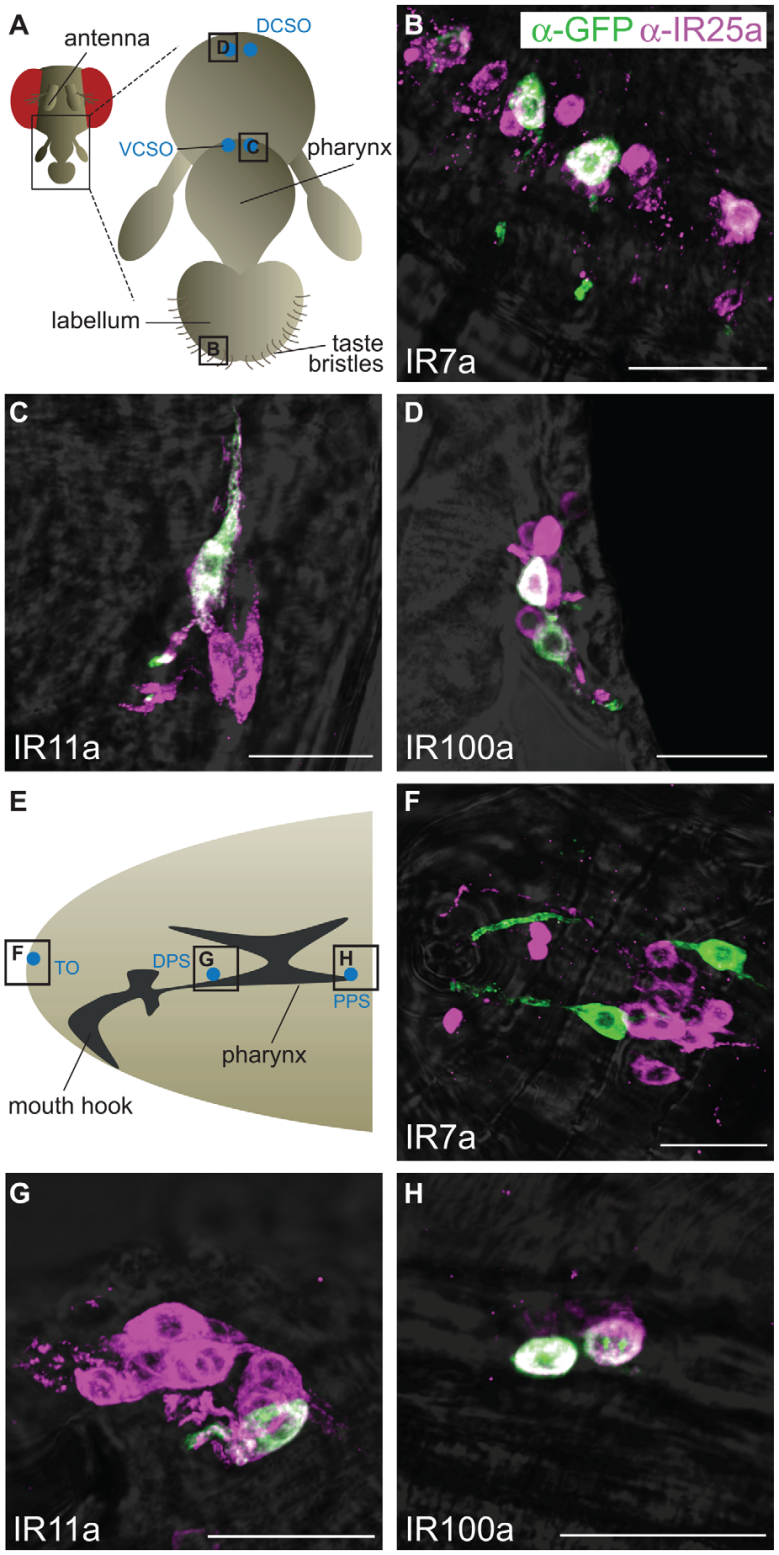
Expression of divergent IRs in *D. melanogaster* adult and larval gustatory organs. Immunofluorescence with anti-GFP (green) and anti-IR25a (magenta) antibodies (overlaid on bright-field images) on whole-mount tissues from animals expressing a membrane targeted GFP reporter transgene (*UAS-mCD8:GFP*) under the control of the indicated *IR promoter-GAL4* driver transgenes. The scale bars represent 20 µm. (A) Schematic of the adult *D. melanogaster* proboscis showing the location of the field of views in (B–D). DCSO: dorsal cibarial sense organ, VCSO: ventral cibarial sense organ. (B) *IR7a-GAL4* drives expression of mCD8:GFP in the labellum. (C) *IR11a-GAL4* drives expression of mCD8:GFP in the VCSO. (D) *IR100a-GAL4* drives expression of mCD8:GFP in the DCSO. (E) Schematic of the *D. melanogaster* larval head showing the location of the field of views in (F–H). TO: terminal organ, DPS: dorsal pharyngeal sense organ, PPS: posterior pharyngeal sense organ. (F) *IR7a-GAL4* drives expression of mCD8:GFP in the TO. (G) *IR11a-GAL4* drives expression of mCD8:GFP in the DPS. (H) *IR100a-GAL4* drives expression of mCD8:GFP in the PPS.

Expression of three of these divergent IR reporters was observed in highly selective populations of neurons in distinct gustatory organs (Figure 5A). In the adult, *IR7a* is expressed in at least eleven neurons in the labellum, a sense organ involved in peripheral taste detection (Figure 5B) [31]. Two reporters labelled neurons in internal sense organs in the pharynx: *IR11a* is expressed in one neuron in the ventral cibarial sense organ and *IR100a* is expressed in two neurons in the dorsal cibarial sense organ (Figure 5C and 5D). These internal pharyngeal neurons are thought to play a role in assessment of ingested food prior to entry into the main digestive system [16]. Expression was not detected in any other neurons or other cell types in the adult head (data not shown), although we cannot exclude expression in other regions of the body. *IR52b* and *IR56a* reporters were not detected in these experiments.

We also examined expression of these reporters at an earlier stage in the *D. melanogaster* life cycle, third instar larvae, which display robust gustatory responses [16]. The same three IR reporters were exclusively detected in unique bilaterally-symmetric larval gustatory organs: *IR7a* was expressed in two neurons in the terminal organ at the periphery, *IR11a* in a single neuron in the ventral pharyngeal sense organ and *IR100a* in two neurons in the posterior pharyngeal sense organ (Figure 5E–5H). Notably, all of these neurons in both adult and larval tissues (except for a single *IR7a*-expressing cell in the terminal organ) co-express IR25a, as revealed by a specific antibody against this receptor (Figure 5) [15]. IR25a is also expressed in several other cells in each of the gustatory organs, which may express other divergent IRs not examined here. Together these results support a role for divergent IRs as taste receptors in distinct taste organs and stages of the *D. melanogaster* life cycle.

### IR evolution on the *Drosophila* phylogeny

To obtain more detailed insights into the processes underlying the expansion and diversification of IR repertoires, we investigated their evolution over a shorter timescale by comparative analysis of *D. melanogaster* with 11 additional sequenced drosophilid species [32]–[33]. The last common ancestor of these drosophilids is estimated to have existed 40 million years ago [34], by contrast to the ∼250 million years since the last common ancestor of *D. melanogaster* and the mosquito *A. gambiae* [35]. Certain species may have diverged much more recently, such as *D. simulans* and *D. sechellia*, whose last common ancestor may have existed only 250,000 years ago [36].

**Figure 6:**
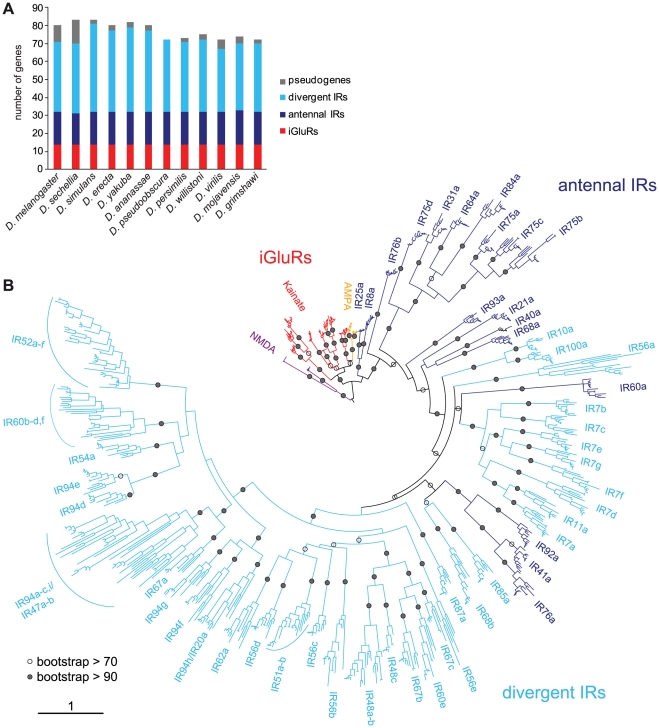
Drosophilid IR repertoires. (A) Histogram of the number of IR and iGluR loci identified in the twelve drosophilid species. (B) Phylogenetic tree of all iGluR and IR genes (excluding pseudogenes and incomplete genes) in the twelve drosophilid species. The tree was constructed using PhyML [76] under the JTT model of substitution and is based on the most conserved columns of an amino acid alignment. Bootstrap values were estimated using an approximate likelihood ratio test and are shown as percentages only for internal nodes. The phylogeny was rooted using the NMDA receptors. The scale bar represents the expected number of substitutions per site.

We used *D. melanogaster* sequences as queries in exhaustive BLAST searches of the drosophilid genomes. Retrieved sequences were manually reannotated to unify gene structure predictions across species and, in some cases, genes were partially resequenced to close sequence gaps or verify them as pseudogenes (see Materials and Methods, Table S3, and Datasets S1 and S2). Although predicted full-length gene sequences could be annotated for most genes, 28 sequences remain incomplete - but assumed in further analysis to be functional - because of a lack of sequence data or difficulty in precise annotation of exons in divergent regions of these genes. Of the 926 drosophilid sequences identified (including those of *D. melanogaster*), 49 genes were classified as pseudogenes because they consisted of only short gene fragments or contained frameshift mutations and/or premature stop codons. We clustered all genes into orthologous groups by examining their sequence similarity, phylogenetic relationships and, in the case of *IR47a*, *IR47b*, *IR47c*, *IR56e* and *IR60f*, their micro-syntenic relationships (Table S1 and Figure 6). For drosophilid species that are most distant from *D. melanogaster*, definition of precise orthologous relationships was not always possible, particularly for groups of closely related IR genes (e.g. IR52a–f, IR60b–f) (Table S1). Orthologous groups were named after their *D. melanogaster* representatives or a logical variant in groups where no *D. melanogaster* gene was identified (see Materials and Methods).

This analysis identified 14 iGluR and 58–69 IR genes in each of the twelve drosophilid species (Figure 6A and Table S1). iGluRs are highly conserved, with a mean amino acid sequence identity of 89±1% s.e.m., and a single representative for each species in every orthologous group. Antennal IRs are also well conserved (mean sequence identity = 76±2%) and amongst these genes we identified only a single pseudogenisation event, in *D. sechellia IR75a*, and a single gene duplication event, of *D. mojavensis IR75d*. By contrast, divergent IRs, though also largely classifiable into monophyletic groups, display a more dynamic pattern of evolution (mean sequence identity = 61±2%), with multiple cases of gene loss, pseudogenisation or duplication (Figure 6 and Table S1).

### Species-specific rates of IR gene loss and gain

**Figure 7:**
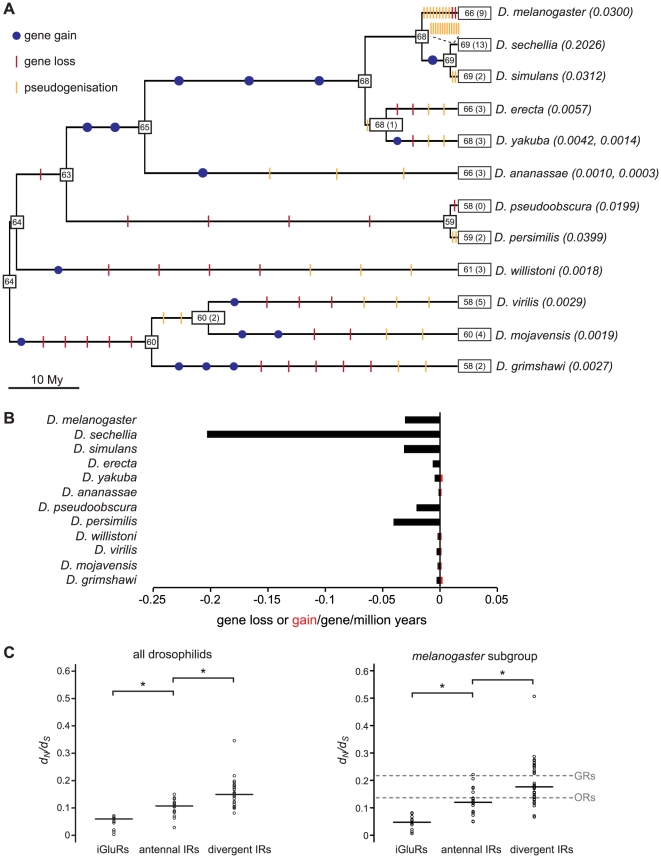
Gene loss and gain and selective pressures in drosophilid IR repertoires. (A) Estimates of the number of IR loci (number of pseudogenes is indicated in parentheses) on internal nodes of the drosophilid phylogeny and gene gain (blue dots), gene loss (red slashes) and pseudogenisation (orange slashes) events on each branch. The gene loss and gene gain rates on the terminal branches are indicated in parentheses after the species names. (B) Histogram of the gene gain (red) and loss (black) rates estimated for the terminal branches of the phylogeny. (C) Distribution and median (horizontal line) of *d*_N_/*d*_S_ rates of iGluR and IR genes estimated for all twelve drosophilid species (left) or five *melanogaster* subgroup species (right). *d*_N_/*d*_S_ values were significantly different between iGluRs, antennal IRs and divergent IRs (p<0.01, Wilcoxon rank-sum tests). In the right-hand plot, the dashed grey lines represent the median values calculated from the *d*_N_/*d*_S_ values for the *melanogaster* subgroup OR and GR genes, as reported in [39]. *d*_N_/*d*_S_ values were significantly different both between antennal IRs and GRs and between divergent IRs and ORs (p<0.01, Wilcoxon rank-sum tests).

We reconciled the gene phylogeny with the drosophilid species phylogeny to estimate the number of IR gene gain and loss events. While this analysis is necessarily constrained by our ability to accurately define gene orthology, we estimated across the entire phylogeny there to be sixteen gene gain events (gene birth rate, B = 0.0006/gene/million years) and 76 gene loss events (gene death rate, D = 0.0030/gene/million years) (Figure 7A, see Materials and Methods). Most (46/76) gene losses are pseudogenisation events, which indicates that many of these events must have occurred relatively recently, as drosophilid species appear to eliminate pseudogenes rapidly from their genomes [37]–[38]. Notably, 13 gene loss events – 12 of which reflect the presence of just one or a small number of premature stop codons or frameshift mutations – occur on the branch leading to the specialist *D. sechellia*. Consequently, the gene loss rate on this branch is remarkably high compared with its generalist sister species *D. simulans* (Figure 7A and 7B).

### Selective forces acting on drosophilid IR genes

We studied the selective forces acting on drosophilid iGluRs and IRs by calculating the ratio of nonsynonymous to synonymous nucleotide substitution rates (*d*_N_/*d*_S_, ω_1_) in these genes from all 12 species. All tested iGluR, antennal IR and divergent IR genes are evolving under strong purifying selection (ω_1_<<1) (Figure 7C, left and Table S4), suggesting that they all encode functional receptors. iGluRs have the lowest estimated *d*_N_/*d*_S_ ratio (median ω_1_ = 0.060), consistent with a conserved role in synaptic communication. Antennal IRs have an intermediate *d*_N_/*d*_S_ ratio (median ω_1_ = 0.107) and divergent IRs the highest (median ω_1_ = 0.149), suggesting that divergent IRs have evolved under weaker purifying selection and/or contain more sites that have been shaped by positive selection. Amongst the IRs, IR25a has the lowest *d*_N_/*d*_S_ ratio (ω_1_ = 0.028), consistent with its high sequence conservation in and beyond drosophilids (Figure 2).

To compare these properties with those of other insect chemosensory receptor families (ORs and GRs) [39], we also calculated *d*_N_/*d*_S_ ratios for IR genes from only the five sequenced species of the *melanogaster* subgroup (*D. melanogaster*, *D. sechellia*, *D. simulans*, *D. erecta* and *D. yakuba*). For this subset of sequences, the relative differences between median *d*_N_/*d*_S_ ratios (ω_2_) for the iGluR and IR gene subfamilies observed with all twelve species was reproduced (Figure 7C, right). The GR gene family has previously been noted to evolve under weaker purifying selection than ORs [39]. Notably, we found that the median *d*_N_/*d*_S_ ratios for antennal IRs (ω_2_ = 0.120) is statistically indistinguishable from that of ORs (ω_2_ = 0.137) (p>0.4, Wilcoxon rank-sum test), and that the median *d*_N_/*d*_S_ ratio of divergent IRs (ω_2_ = 0.176) is statistically indistinguishable from that of GRs (ω_2_ = 0.217) (p>0.5, Wilcoxon rank-sum test). Thus, the selective forces acting on the IR receptor gene subfamilies parallel those on the ORs and GRs and appear to correlate with their putative distinct chemosensory functions in olfaction and gustation (Figure 7C, right). The reason for this difference is unknown, but might reflect reduced evolutionary constraints on co-expressed and partially redundant taste receptor genes or selection for higher diversity in taste receptor sequences to recognise more variable non-volatile chemosensory ligands in the environment.

Most residues of IR proteins can be expected to have evolved under purifying selection to maintain conserved structural and signalling properties, which may mask detection of positive selection (ω>1) at a small number of sites that contribute to their functional diversity. To obtain evidence for site-specific selection we applied site class models M7 and M8 in PAML to analyse 49 sets of orthologous IR genes of the six species of the *melanogaster* group. This test did not identify any sites significantly under positive selection after Bonferroni correction (Table S4), a result consistent with orthologous IR genes having the same function across drosophilids.

Site-specific positive selection may be more easily detectable in relatively recent IR gene duplicates potentially undergoing functional divergence. We therefore analysed the sole duplication of an antennal IR, *IR75d.1* and *IR75d.2* in *D. mojavensis*. Assuming an estimated divergence time of 35 My between *D. virilis* and *D. mojavensis* [40], and based on analysis of *d*_S_ of *IR75d* genes in these species (see Materials and Methods), we estimated this duplication to have occurred relatively recently, approximately 2.6–5.1 My ago. Using a branch-site test we identified two sites (p<0.05) that have evolved under positive selective pressure, where DmojIR75d.1 and DmojIR75d.2 appear to contain the ancestral and derived residues, respectively: DmojIR75d.2-S670 maps to the third transmembrane domain and DmojIR75d.2-Q365 maps to the putative ligand binding domain. Functional characterisation of these variant receptors will be required to determine their significance.

### Expansion of the IR repertoire by gene duplication and retroposition

**Figure 8:**
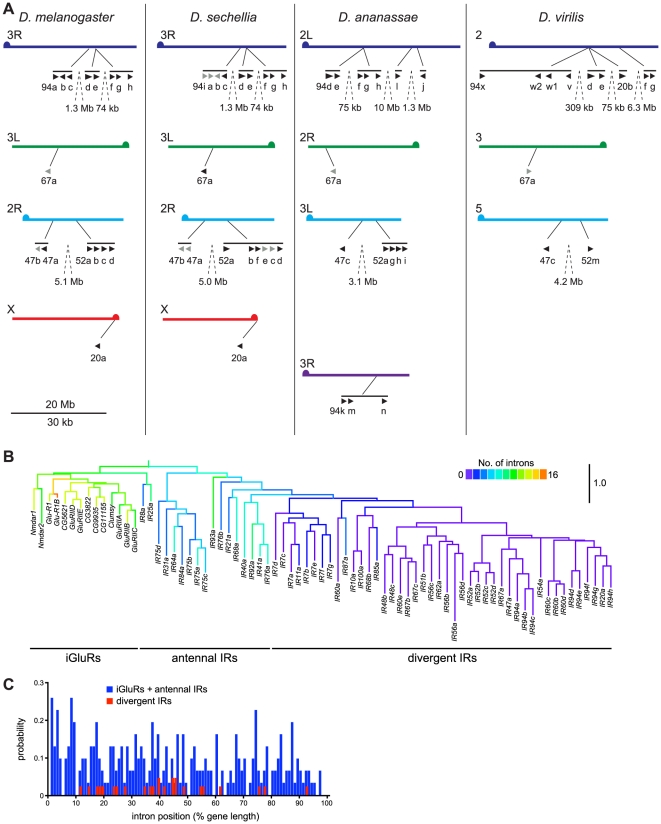
Mechanisms of IR repertoire expansion. (A) Genomic location of the IR genes (black arrowheads; pseudogenes in grey) belonging to the IR94 and IR52 clades in *D. melanogaster*, *D. sechellia*, *D. ananassae* and *D. virilis*. Equivalent chromosome arms (Muller elements) (labelled on the left of each chromosome arm) between the species are indicated by colour and horizontal alignment [93]. Tandem arrays of genes are indicated by horizontal black lines, and the distances between close arrays are shown. The “IR” and some number prefixes for gene names are omitted in clusters where space is limiting. The scale bar represents 20 Mb for the chromosomes and 30 kb for gene lengths and distances between genes within the same tandem array. (B) Phylogenetic tree of *D. melanogaster* iGluRs and IRs, in which branches are colour-coded by the number of introns in each extant gene sequence or predicted ancestor. The tree was built with RAxML under the WAG model of substitution, with 1000 bootstrap replicates, and the colours representing intron numbers were inferred and displayed with Mesquite. Pseudogenes were excluded from this analysis. The scale bar represents the expected number of substitutions per site. (C) Histogram illustrating the distribution of intron positions as a percentage of protein length for iGluRs and antennal IRs (blue) and divergent IRs (red). Each bar represents the probability of occurrence of an intron at a given percentile of the protein.

From potentially one ancestral IR, what genetic processes underlay the generation of large repertoires of IR genes? We initially sought evidence for these mechanisms through analysis of the *D. melanogaster* IR family. Several monophyletic groups of IR genes exist in clusters in the genome suggesting an important role of gene duplication by non-allelic homologous recombination. For example, eight divergent IRs of the IR94 orthologous groups are located in three close, but separate, tandem arrays on chromosome arm 3R (Figure 8A). Other genes in the same clade are also found scattered on other chromosome arms (X, 2R, 3L) (Figure 6 and Figure 8A), indicating that interchromosomal translocation has also occurred frequently, most likely both during and after formation of the tandem arrays. Similar patterns are observed in the orthologous/paralogous sequences of these IRs in other drosophilid species (Figure 8A), as well as for other IR clades (data not shown). These features are also observed in IR repertoires in other insects, although incomplete genome assembly prevented a more precise analysis. For example, in *Aedes aegypti* the 23 IR7 clade members are found in arrays of 1, 1, 2, 5, 7 and 7 genes on 6 different supercontigs (data not shown).

We also noticed an unusual pattern in *D. melanogaster* IR gene structures, in which antennal IRs (as well as iGluRs) contain many (4–15) introns, while the vast majority of divergent IRs are single exon genes (Figure 8B). Drastic intron loss in multigene families is a hallmark of retroposition, where reverse-transcription of spliced mRNAs from parental, intron-containing genes and reinsertion of the resulting cDNA at a new genomic location may give rise to a functional, intronless retrogene [41]. The few introns that are present in these IRs in *D. melanogaster* have a highly biased distribution towards the 5′ end of the gene (19/25 introns in the first 50% of IR gene sequences) (Figure 8C), which is characteristic of recombination of partially reverse-transcribed cDNAs (a process which initiates at the 3′ end) with parental genes [42]. Sequence divergence of IRs prevented us from identifying parental gene-retrogene relationships. Nevertheless, these observations together suggest that divergent IRs arose by at least one, and possibly several, retroposition events of ancestral antennal IRs. Once “born”, single exon IRs could presumably readily further duplicate by non-allelic homologous recombination.

## Discussion

### A model for iGluR and IR evolution

**Figure 9:**
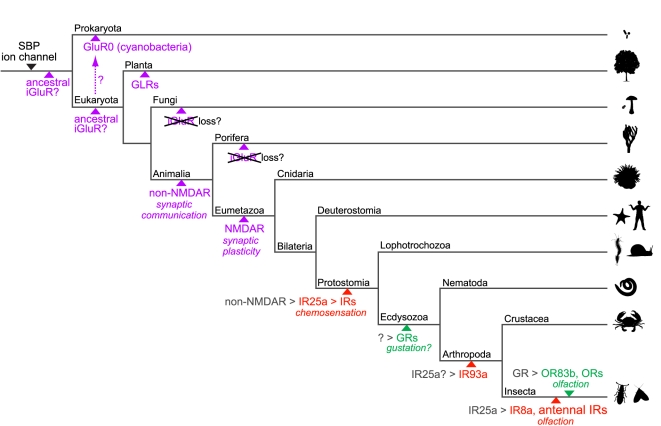
A model for the evolution of iGluRs and IRs. Schematic phylogenetic tree highlighting the branches along which specific gene families or genes appeared with their putative functions, inferred from their presence or absence in sequenced genomes of extant species (see Figure 1). Solute binding proteins (SBPs, which exhibit the same protein fold as the iGluR/IR amino terminal domain and ligand-binding domain) and ion channels were likely present in primitive life forms as related protein domains exist in Eukaryota, Bacteria and Archaea [94]. iGluRs are shown in purple, IRs in red and insect GRs and ORs in green. Various speculative models for the origins of iGluRs are shown. Putative genetic ancestors from which IRs, GRs and ORs derived are shown in grey followed by a “>” symbol. The resolution of the phylogeny is necessarily biased towards invertebrate lineages and branch lengths contain no temporal information.

Our comprehensive survey and phylogenetic analysis of iGluR/IR-like genes permits development of a model for their evolution (Figure 9). The shared, unusual “S1-ion channel-S2” domain organisation of prokaryotic GluR0 and eukaryotic iGluRs is suggestive of a common ancestor of this family by fusion of genes encoding the separate domains that were present in very early life forms (Figure 9) [11]. However, we have found prokaryotic glutamate receptors in only a very small number of bacterial species. Thus, if an iGluR evolved in the common ancestor of prokaryotes and eukaryotes, it must have subsequently been lost in a large number of prokaryotic lineages. It is possible, therefore, that iGluRs only originated in eukaryotes and were acquired by certain prokaryotic species by horizontal gene transfer [43]. If the latter hypothesis is true, the presence of closely related iGluRs in both plants and animals implies their early evolution within eukaryotes, potentially in the last common eukaryotic ancestor [44]. However, the absence of iGluRs in sponges and all examined unicellular eukaryotes raises the alternative possibility that animal and plant receptors evolved independently, or were acquired by horizontal transmission, perhaps from prokaryotic sources. Whatever the precise origin of iGluRs in animals, their subsequent divergence into AMPA, Kainate and NMDA subfamilies also occurred early, although variation in the size of these subfamilies suggests continuous adaptation of the synaptic communication mechanisms they serve to nervous systems of vastly different complexities.

Several outstanding issues regarding IR evolution can now be addressed. First, we have shown that the IRs were very likely to have been present in the last common ancestor of Protostomia, an estimated 550–850 million years ago [20]. *IR25a* represents the probable oldest member of this repertoire and conservation of chemosensory organ expression of *IR25a* orthologues in molluscs, nematodes, crustaceans and insects strongly suggests that this receptor may have fulfilled a chemosensing function in the protostome ancestor.

Second, the apparent absence of IRs in Deuterostomia suggests the parsimonious model that IRs evolved from an animal iGluR ancestor rather than representing a family of chemosensing receptors that was present in a common ancestor of Animalia and lost in non-protostomes. Our phylogenetic and gene structure analysis suggests that *IR25a* may have derived from a non-NMDA receptor gene. The transition from an iGluR to an IR may not have involved drastic functional modifications: both receptor types localise to specialised distal membrane domains of neuronal dendrites (post-synaptic membranes and cilia, respectively) and, in response to binding of extracellular ligands, depolarise these domains by permitting transmembrane ion conduction which in turn induces action potentials [45]. Thus, it is conceivable that IRs arose simply by a change in expression of an iGluR from an interneuron (where it detected amino acid signals from a pre-synaptic partner) to a sensory neuron (where it could now detect chemical signals from the external environment).

Third, our analyses of IR repertoires across both divergent and relatively closely related species provide insights into the mechanistic basis for the expansion and functional diversification of the IR repertoire. Gene duplication by non-allelic homologous recombination is a widespread mechanism for growth of most multigene families in chemosensory systems [46], and this is also true for the IRs. Our implication of retroposition as a second mechanism in the evolution of IR repertoires offers two advantages for functional diversification. First, by arising from random re-insertion of reverse transcribed copies of parental genes, retrogenes normally lack endogenous promoter sequences, and can therefore potentially acquire novel expression patterns from genomic sequences flanking their insertion site that are distinct from their parental ancestor [41]. Indeed, in *D. melanogaster*, retrogene or retrogene-derived IRs - the divergent IRs - are apparently no longer expressed in antennal neurons like their ancestors, but instead in gustatory (and perhaps other) tissues. Second, release from the evolutionary constraints of the preservation of splicing signals near exon boundaries may have contributed to the more rapid divergence of the protein sequences of these intronless IRs [47].

Analysis of IR repertoires across the well-defined drosophilid phylogeny provides clear evidence for a birth-and-death model of evolution, in which, following gene duplication, individual family members progressively diverge in sequence and, in some cases, are lost by pseudogenisation and/or deletion [48]–[49]. Differential rates of these processes will ultimately shape the precise IR repertoire of an individual species (discussed below).

### Evolutionary and functionally distinct IR subfamilies: olfactory and gustatory receptors, and ligand-binding receptors and co-receptors

Our molecular evolutionary analysis has distinguished two subfamilies in the IR repertoire: conserved, antennal IRs and the species-specific, divergent IRs. Their distinct evolutionary properties may correspond to fundamental functional differences, as we provide here the first evidence, to our knowledge, for expression of divergent IR subfamily members in subsets of neurons in both peripheral and internal gustatory organs at both adult and larval stages of *D. melanogaster*. The selective and non-overlapping expression patterns observed in the small sample of IR genes examined indicate that a large fraction of the divergent IR repertoire may be expressed in gustatory neurons. It is also possible that some of these IRs may be expressed in non-chemosensory tissues. Although subsets of GR genes have been implicated in the detection of sweet or bitter compounds in peripheral taste bristles in *D. melanogaster* [31], a comprehensive understanding of the physiological breadth and molecular logic of taste detection is lacking. Our results introduce further complexity into the molecular mechanisms of taste detection and demand comprehensive and comparative expression and functional analysis of divergent IRs and GRs in this sensory system.

Although many gustatory-expressed divergent IRs in *D. melanogaster* are recently derived in drosophilids, the ancestral chemosensory function of IRs is likely to be not in the detection of airborne volatiles but rather water-soluble, non-volatile compounds, as the last common ancestor of Protostomia was probably aquatic. Indeed, the strikingly similar expression of IR genes in internal pharyngeal neurons in *D. melanogaster* and *C. elegans* suggests a conserved role for these receptors in sensing chemical signals from ingested food. In this light, the derivation of IRs from receptors detecting amino acid-related neurotransmitters invites the attractive hypothesis that ligands for these gustatory IRs (as well as species-specific IRs in other protostomes) are also amino acids. Almost nothing is known about sensory responses to this class of chemical signals in *D. melanogaster*, despite their vital importance for normal insect physiology and metabolism [50], but amino acids are chemosensory stimulants in other insects, lobsters and molluscs [51]–[53].

Our evolutionary and expression studies have highlighted IR25a as an atypical member of the repertoire, displaying deep conservation and broad expression in many olfactory and gustatory neurons. While we cannot exclude the possibility that IR25a recognises a specific chemical ligand, co-expression of this receptor with other cell-type specific IRs favours a model in which this acts as a co-receptor, analogous both to the heteromeric assembly of iGluR subunits into functional complexes [1], as well as to the pairing of ligand-specific ORs with the common OR83b co-receptor [54]–[55]. An insect- and antennal-specific homologue of IR25a, IR8a, may play a similar role specifically for olfactory IRs.

### A common insect nose and species-specific IR repertoires

In addition to IR25a and IR8a, many other *D. melanogaster* antennal IRs are highly conserved in insects, both in sequence and expression pattern. These properties contrast starkly with the insect OR repertoires, which probably evolved only in terrestrial insects [56], and which contain only one member displaying orthology across multiple orders, the atypical OR83b co-receptor [57]. ORs are an expanded lineage of the ancestral GR repertoire whose evolutionary origins are unknown [56]. Homologues of GR genes exist in *D. pulex* and *C. elegans* [56], [58], but in the latter species these receptors may not be involved in chemosensation [59]–[60]. These observations suggest that, in insects, the IRs represent the first olfactory receptor family, whose members were fixed functionally early in their evolution to detect olfactory stimuli that are important for all species of this animal class. Consistent with this, the antenna of the mayfly *Rhithrogena semicolorata* – an insect belonging to the Paleoptera and not the Neoptera that encompasses all species described here – bears coeloconic sensilla (potentially housing IR-expressing neurons) but not trichoid or basiconic sensilla (which house OR-expressing neurons in all other insects examined) [61]. Available data on ligands for IR sensory neurons - and the role of specific IRs within these neurons - are limited, but include stimuli such as carboxylic acids, water and ammonia, which are known to be physiologically and behaviourally important in many insect species [62]. ORs, by contrast, may be primarily dedicated to detection of species-specific odour cues. In this light, the IRs are attractive molecular targets for novel, broad-spectrum chemical regulators of insect odour-driven behaviours, with applications in the control of disease vectors, such as mosquitoes, and agricultural pests.

Given the general conservation of the antennal IRs, what is the significance of the more recently evolved, species-specific variation in this family of chemosensory receptors? It is particularly informative to consider this question in the evolutionarily closely related drosophilid species. These display prominent differences in their global geographical distribution and chemosensory-driven behaviours [63]–[64], and include both generalists, which feed and breed on a wide range of substrates, and specialists, which have highly restricted ecological niches. The chemical ecology is best-understood for *D. sechellia*, a species endemic to the Seychelles that utilises the acid-rich fruit of *Morinda citrifolia* as its sole food source and oviposition site, a remarkable specialisation as this fruit is repulsive and toxic for other drosophilids [64]–[65]. Genetic hybrids between *D. sechellia* and *D. simulans* indicate that host specialisiation is due to loss-of-function mutations, rather than gain of new chemosensory perception abilities [65]. The accelerated rate of IR gene loss in *D. sechellia* compared to its sibling *D. simulans* (and other drosophilids) bears the hallmark of genetic adaptation of this chemosensory repertoire to the restricted host fruit. Notably, one of the *D. sechellia* pseudogenes is *IR75a*, an antennal IR expressed in a neuron responsive to several acids [62]. Thus, *DsecIR75a* represents an interesting gene whose mutation may be directly linked to host specialisation of this species. Future study of this receptor, and other species-specific IRs, may offer novel models to link genetic changes with phenotypic adaptation during animal evolution.

### Genetic insights into the origins of animal olfactory systems

Finally, our results may shed light into the outstanding question of the evolutionary origin of animal olfactory systems. Common neuroanatomical features have long been appreciated in animal olfactory circuitry, notably glomeruli, which represent sites of synaptic connection of OSNs of identical molecular and physiological specificity with second order neurons [66]. Whether these represent homologous or analogous structures across phyla is unclear. Revelations of fundamental distinctions in the structure, function and regulation of mammalian and insect ORs support a theory of convergent evolution of the neuronal circuits in which these receptors act [67]–[68].

Our demonstration that most, if not all, insect olfactory systems comprise two molecularly distinct receptor families, the ORs and IRs, indicates that the evolution of receptor repertoires can be uncoupled from a presumed common origin of the OR and IR neuronal circuits within the insect ancestor. Thus, during a significantly greater timescale across animal phyla, profound molecular differences between olfactory receptor genes do not necessarily imply distinct evolutionary origins of the neuronal circuitry in which they are expressed. Our discovery of IRs in mollusc olfactory organs reveals this to be an interesting potential “hybrid” organism in olfactory system evolution. The *A. californica* rhinophore and oral tentacle also express a large family of GPCR-family candidate chemosensory receptors, belonging to the same Rhodopsin superfamily as vertebrate ORs [21]. The co-existence of both insect-like and vertebrate-like olfactory receptors in this species provides evidence for the occurrence of an evolutionary transition between these distinct olfactory receptor families. Thus, while extant animal olfactory systems display an enormous diversity in their receptor repertoires, there may remain - perhaps unexpectedly - a sufficient genetic trace within receptor gene families themselves to open the possibility of a common evolutionary origin of this sensory system.

## Materials and Methods

### Gene identification and annotation

#### Eukaryota (non-drosophilids)

Genomic and available annotated protein databases for each eukaryotic species were downloaded from the sources described in Table S2 (spring 2009 versions). Prokaryotic genome and protein sequences were downloaded from NCBI. We built and calibrated an HMM with HMMER [69] for iGluR/IR gene identification by adding sequences of the *D. melanogaster* PF00060 domain (iGluR ligand-gated ion channel) to those of the PF00060 domains from the Pfam database [17]. This HMM (LC05) was used to screen protein databases using HMMER. For each species, all significant hits (HMMER E value <e-5) were subsequently used, in addition to *D. melanogaster* iGluR and IR sequences [15], as queries in exhaustive PSI-BLAST searches with standard parameters until convergence. All identified sequences (below an arbitrary threshold E value <e-5) were then used as queries in TBLASTN searches of genomic DNA databases. For each DNA hit (E value <e-3), we analysed a genomic region of approximately 20 kb spanning this sequence for the presence of a *bona fide* iGluR or IR gene, by using the LC05 HMM and homology analysis with *D. melanogaster* iGluRs and IRs to annotate exons in these regions using GeneWise [70]. Predicted proteins were verified by analysing the number and placement of transmembrane segments using the TMHMM Server v2.0 [71], and domain organisation using the Pfam database. Most annotated sequences (Datasets S1 and S2) appear to be incomplete at their 5′ ends as they do not encode N-terminal signal sequences, as determined by analysis with SignalP 3.0 [72], and we were normally not able to annotate this part of the protein with confidence. However, as this region is highly divergent in amino acid sequence, its absence is likely to have little influence on our phylogenetic analyses.

#### Drosophilids

*D. melanogaster* iGluR and IR sequences were used as queries in exhaustive PSI-BLAST and TBLASTN searches of the genome assemblies described in Table S2. PSI-BLAST was carried out for 20 iterations or until no new sequences with an E value <e-3 were recovered. For genes that were apparently missing in some species, we used manual syntenic analysis to determine whether this represented a real absence from the genome. Genes were manually reannotated to ensure the presence of appropriate structural features as described above, as well as reasonable splice site signals and start/stop codons. Missing or mis-annotated exons in one species were usually easily corrected by comparison with homologous sequences in other species. Genes containing nonsense mutations were manually resequenced (see below) to confirm or refute their annotation as pseudogenes (Table S3). We also resequenced parts of genes where there were gaps in the genome assembly (Table S3).

### Phylogenetic analyses

#### Protein tree building

The amino acid sequences of the selected iGluRs/IRs were aligned with PROBCONS [73] and examined in Jalview [74]. The alignments were cleaned manually to obtain final high-quality alignments of 150–300 residues, depending on the sequences analysed (see Dataset S3 for all alignments pre- and post-cleaning). We used ProtTest [75] to assess the best model of substitution to infer the phylogeny. The trees were then calculated with PhyML [76] or RAxML [77] and viewed and graphically edited with FigTree (tree.bio.ed.ac.uk), Mesquite [78] or iTOL [79]. For trees of drosophilid iGluRs/IRs, pseudogenes and incomplete genes were excluded from alignments, and we applied the JTT model of amino acid substitution in PhyML. Bootstrap values were estimated using an approximate likelihood ratio test.

#### Character matrix tree building

Selected protein sequences were aligned using MUSCLE [80] and the positions of introns were reported on the alignment. A character matrix was built according to the presence of introns at each potential intronic site. The tree was built using the PARS software from the PHYLIP package (evolution.genetics.washington.edu/phylip.html).

#### Orthology determination

Genes were defined as orthologous when they were best reciprocal BLAST hits and when they grouped in the same clade in phylogenetic trees. Because we could not unambiguously assign orthologues to some IRs, we classified those genes as members of larger orthologous groups encompassing several members in some species.

### Gene and protein nomenclature

IR genes were named according to a unified nomenclature system based upon a foundation of the cytologically derived *D. melanogaster* IR gene names [15]. Receptor names are preceded by a four-letter species abbreviation consisting of an uppercase initial letter of the genus name and three lower case initial letters of the species name (e.g. *Anopheles gambiae* = *Agam*; *Daphnia pulex* = *Dpul*). Orthologues of *D. melanogaster* sequences are given the same name (e.g. *CquiIR25a*, *AcalIR25a*). If multiple copies of an orthologue of a *D. melanogaster* gene exist for a species (based on sequence, not function), they are given the same name followed by a point and a number (e.g. *ApisIR75d.1*, *ApisIR75d.2*). If several in-paralogues exist both in *D. melanogaster* and other species, these are all given the same number (indicating their grouping within a common clade), but different final letterings. For novel, species-specific IRs, we defined new names numbering from 101 upwards to avoid confusion with *D. melanogaster* gene names, which number up to IR100a. For species-specific IRs that form monophyletic clades and had high (>60%) amino acid identity, we gave these the same name with an additional number suffix after a point (e.g. *AaegIR75e.1*, *AaegIR75e.2*). We did not rename genes with previously published names (e.g. *C. elegans* GLR-7 and GLR-8 [9]).

For vertebrate iGluRs, we used the NC-IUPHAR nomenclature [81]: each species name is followed by “Glu”, a letter representing the subtype of the receptor (K for Kainate, A for AMPA and N for NMDA), and a number, reflecting predicted orthology with mammalian iGluRs. We did not name (or rename) invertebrate iGluRs in this study, except for newly predicted gene sequences (Table S3), where logical variants of NC-IUPHAR nomenclature were assigned.

### Evolutionary analysis

#### Gene birth and death rate estimation

To estimate the gene birth and death rates of IRs on the drosophilid phylogeny we used the gene numbers listed in Table S1. Incomplete genes (i.e. genes for which we could not annotate full-length sequences because of lack of sequence data) were classified as present. To estimate the number of gene gain and loss events for each orthologous group we estimated gene numbers on internal branches using a maximum likelihood method [82] implemented in the software CAFÉ [83]. These numbers were then summed to estimate the number of IR gene gains and losses on each branch of the phylogeny. The divergence times for the species tree were taken from the published estimates [40], [84]. The gene birth and death rates per million years on the terminal branches were calculated as number of gene losses or gene gains divided by the number of genes on the respective internal node divided by the length in million years of the respective terminal branch. The gene death rates, D, averaged over the whole species tree were calculated as in [85]: 
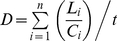
, where *n* is the number of branches in the tree, *L_i_* is the number of gene losses on branch *i*, *C_i_* is the number of gene copies at the internal node of branch *i* and *t* is the total time of the phylogeny. For the estimation of the gene gain rate, B, *L_i_* was replaced by the numbers of gene gains, *G_i_*, on branch *i*.

#### Analysis of selective forces

We inferred the *d*_N_/*d*_S_ ratio (ω) by maximum likelihood as implemented in PAML [86]. All PAML analyses were run three times using different input parameters to avoid local optima. To create multiple sequence alignments of orthologous genes, we first aligned the amino acid sequences using MUSCLE. Pseudogenes and incomplete genes were avoided in these analyses, and if genes had multiple annotated isoforms we used only those conserved with the other species. The resulting alignments were then used to guide the nucleotide coding region alignments using custom-written software [87]. Columns with gaps were omitted for the *d*_N_/*d*_S_ calculations. For all analyses, we assumed the topology illustrated in Figure 7A. We applied model M0 to estimate the global selective pressure acting on the IR and iGluR genes. To compare our data with a previous analysis of drosophilid ORs [39], we applied a branch model to estimate the global selective pressure acting on the IR and iGluR genes. In this model, one *d*_N_/*d*_S_ ratio was assigned to the five *melanogaster* subgroup species and one ratio was assigned to *D. ananassae* (model = 2, NSsites = 0). The *D. ananassae* ratio was then discarded to leave one *d*_N_/*d*_S_ ratio depicting the selective pressure acting on the respective gene in the *melanogaster* subgroup.

To identify positively selected sites we applied models M7 (beta) and M8 (beta & ω) in PAML and compared them using a maximum likelihood ratio test (LRT). If M8 fitted the data significantly better than M7, we applied a Bayes Empirical Bayes (BEB) estimation method as implemented in PAML to identify the sites that are estimated to be under positive selection.

We applied another test to analyse the duplication of *IR75d* in *D. mojavensis*. To test if residues of these genes evolved under positive selective pressure, we first compared a model that assigns one single *d*_N_/*d*_S_ ratio to all branches with a model that assigns one additional ratio to the branches following the duplication. If this second model fitted the data significantly better than the first model we used branch-site model A (model = 2, NSsites = 2) with ω = 1 fixed on the branches after the duplication as null model and compared it to this same model A but allowing ω>1 on the branches following the duplication. To estimate the age of the *IR75d* duplication in *D. mojavensis*, we applied model M0 to estimate *d_S_* on the branch before the duplication and on the two branches after the duplication. By relating these *d_S_* values to each other and assuming a divergence time of 35 My between *D. mojavensis* and *D. virilis*, we obtained two estimates of the timing of the duplication event.

### Re-sequencing of drosophilid IR genes

Genomic DNA was extracted from the sequenced drosophilid genome strains (obtained from the *Drosophila* Species Stock Center, University of California-San Diego) using a standard DNA extraction protocol. PCR primers were designed to amplify ∼500 bp regions covering putative nonsense or missense mutations or spanning gaps in the genome sequence (oligonucleotide sequences are listed in Table S5). PCR amplifications were performed using Taq DNA Polymerase (PEQLAB Biotechnologie GmbH) in a MasterCycler Gradient Thermocycler (Eppendorf) with the following programme: 95°C for 3 min, 35 cycles of (95°C for 30 sec, 55°C for 1 min, 72°C for 1 min) and 72°C for 10 min, with minor modifications of annealing temperature and elongation times for different primer pairs and amplicon sizes. Products were gel purified (Machery-Nagel) and sequenced with BigDye Terminator v3.1 according to the manufacturers' protocols.

### Reverse-transcription PCR

Insects: total RNA was extracted from hand-dissected tissues of wildtype *A. mellifera* and *D. melanogaster* (*w^1118^* strain) using the RNeasy Mini Kit (Qiagen), and reverse-transcribed using oligo-dT primers and the SuperScript III First-Strand Synthesis System (Invitrogen). Genomic DNA was extracted using standard procedures. Primers were designed to amplify short regions overlapping an intron, if possible at the 3′ end of the coding sequence (Table S5). PCR product amplification and purification were performed as described above and sequenced to verify their identity. Multiple independent cDNA preparations were analysed for each primer pair.

#### *Aplysia*

Mature *Aplysia dactylomela* (100–300 g) were collected from Kings Beach, Caloundra, Queensland, Australia. Animals were anaesthetised in 337 mM MgCl_2_ equivalent to 50% of their weight. Tissues were removed and snap frozen in liquid nitrogen for RNA isolation. Adult *Aplysia californica* (100–500 g) were obtained from Marine Research and Educational Products (Escondido, CA, USA), and the rhinophore was removed and stored in RNAlater (Qiagen). Total RNA was extracted from samples using TRI Reagent (Sigma). One µg of total RNA was treated with DNase I (Invitrogen) and cDNA was synthesised from 0.5 µg DNase-treated RNA using 200 ng random pentadecamers and the Superscript III Reverse Transcriptase System (Invitrogen). No-RT controls were also carried out for each RNA sample using 0.5 µg DNase-treated RNA to confirm the absence of genomic DNA contamination. PCR amplification using primer pairs for individual *Aplysia* IRs or for a β-actin control (Table S5) were performed using RED*Taq* DNA polymerase (Sigma) according to the manufacturer's protocol.

### Construction of *IR-GAL4* transgenes

Primers were designed to amplify putative promoter regions from *Oregon-R D. melanogaster* genomic DNA with flanking restriction sites, extending from immediately upstream of the predicted start codon to the following 5′ extents: *IR7a* (2318 bp), *IR11a* (2099 bp), *IR52b* (446 bp), *IR56a* (2400 bp) and *IR100a* (512 bp) (Table S5). Gel purified PCR products were T:A cloned into *pGEM-T Easy* (Promega), end-sequenced, and sub-cloned into a *pGAL4-attB* vector, comprising the *GAL4 ORF-hsp70-3′UTR* in the *pattB* vector [30]. These constructs were integrated into the attP2 landing site [88], by standard transformation procedures (Genetic Services, Inc.). *IR-GAL4* transgenic flies were double-balanced and crossed with flies bearing a *UAS-mCD8:GFP* transgene [89] to visualise driver expression.

### Histology

#### RNA *in situ* hybridisation on *Aplysia*

a 743 bp region of *A. dactylomela IR25a* cDNA was amplified and cloned into *pGEM-T* (Promega) as a template for synthesis of sense and antisense digoxigenin-labelled RNA probes (Roche). *In situ* hybridisation on 12 µm rhinophore cryosections was performed essentially as described [90]. Sections were photographed using an Olympus BX60 with Nomarski optics and a Nikon Digital Sight DS-U1 camera.

#### Immunofluorescence on larval and adult *Drosophila*

Third instar larvae were placed in a Petri dish containing 1×PBS/0.1% Triton (P/T) and their head regions containing chemosensory organs were removed with forceps. For adults, probosci were pulled off the head with forceps and the labellum and the more proximal parts separated. Dissected tissues were placed in a 1.5 ml microcentrifuge tube and fixed in 4% PFA in 1×PBS for 1 hour at 4°C, washed 3×10 minutes in P/T, blocked for 30 minutes in 5% heat-inactivated goat serum in P/T (P/T/S) and incubated overnight at 4°C with mouse anti-GFP (Invitrogen) and rabbit anti-IR25a [15], both diluted to 1∶500 in P/T/S. Tissues were washed and blocked as above and incubated with Alexa488-anti mouse and Cy3-anti rabbit secondary antibodies (Milan Analytica AG), both diluted to 1∶500 in P/T/S for 2 hours at room temperature. Samples were mounted on glass slides with 100 µl Vectashield. Images were collected with a Zeiss LSM 510 Meta upright confocal microscope (Zeiss, Oberkochen, Germany), using a Plan-APOCHROMAT 63×/1,40 Oil DIC objective.

## Supporting Information

Dataset S1 iGluR and IR predicted protein sequences. Sequences are in FASTA format. The header line of each sequence displays i) the new sequence name (except for previously annotated non-vertebrate iGluRs), ii) the old sequence name (for previously annotated sequences) and, in some cases, iii) comments, separated by spaces. Internal stop codons and frameshifts are indicated by an ‘X’. Unknown residues (due to gaps in genomic sequence data) are indicated by an ‘x’.
(1.16 MB TXT)

Dataset S2 iGluR and IR predicted transcripts. Sequences are in FASTA format. The header line of each sequence displays the new sequence name, except for previously annotated non-vertebrate iGluRs.
(3.40 MB TXT)

Dataset S3 Alignments used for phylogeny. This folder contains the multiple sequence alignments used for phylogenetic analyses, before and after alignment cleaning in FASTA and PHYLIP format, respectively, as well as the intron alignment file used in Figure 2D.
(1.21 MB ZIP)

Table S1 Drosophilid iGluR and IR repertoires.
(0.04 MB XLS)

Table S2 Sources of eukaryotic genomic and protein sequence data.
(0.70 MB DOC)

Table S3 Nomenclature of newly annotated and previously identified iGluR and IR genes.
(0.19 MB XLS)

Table S4 Nonsynonymous to synonymous substitution rates of IR genes.
(0.03 MB XLS)

Table S5 Oligonucleotides.
(0.05 MB XLS)
