# Supplementary material for: Ancient Protostome Origin of Chemosensory Ionotropic Glutamate Receptors and the Evolution of Insect Taste and Olfaction
Source: PLoS Genet. 2010 Aug 19;6(8):e1001064. doi: 10.1371/journal.pgen.1001064 (PMC2924276; doi:10.1371/journal.pgen.1001064)
Supplement: Table S2 — Sources of eukaryotic genomic and protein sequence data. (0.70 MB DOC) [file pgen.1001064.s005.doc]

**Table S2. Sources of eukaryotic genomic and protein sequence data.**

| Species | Genome version | Proteome version | URL | Reference |
| --- | --- | --- | --- | --- |
| *D. melanogaster* | 4.55 | 5.16 | [http://flybase.org](http://flybase.org/) | [1] |
| *D. sechellia* | r1.3 | r1.3 | [http://flybase.org](http://flybase.org/) | [2] |
| *D. simulans* | r1.3 | r1.3 | [http://flybase.org](http://flybase.org/) | [2] |
| *D. erecta* | r1.3 | r1.3 | http://flybase.org | [2] |
| *D. yakuba* | r1.3 | r1.3 | http://flybase.org | [2] |
| *D. ananassae* | r1.3 | r1.3 | http://flybase.org | [2] |
| *D. pseudoobscura* | r2.3 | r2.3 | http://flybase.org | [3] |
| *D. persimilis* | r1.3 | r1.3 | http://flybase.org | [2] |
| *D. willistoni* | r1.3 | r1.3 | http://flybase.org | [2] |
| *D. virilis* | r1.2 | r1.2 | http://flybase.org | [2] |
| *D. mojavensis* | r1.3 | r1.3 | http://flybase.org | [2] |
| *D. grimshawi* | r1.3 | r1.3 | http://flybase.org | [2] |
| *A. aegypti* | 1.0 | 1.54 | [http://vectorbase.org](http://vectorbase.org/) | [4] |
| *C. quinquefasciatus* | 1.0 | 1.2 | [http://vectorbase.org](http://vectorbase.org/) | [5] |
| *A. gambiae* | 3.54 | 3.53 | [http://vectorbase.org](http://vectorbase.org/) | [6] |
| *B. mori* | 1.0 | 1.0 | [http://silkworm.genomics.org.cn](http://silkworm.genomics.org.cn/) | [7] |
| *T. castaneum* | 3.0 | 3.0 | [http://www.beetlebase.org](http://www.beetlebase.org/) | [8] |
| *A. mellifera* | 4.0 | 4.0 | [http://www.beebase.org](http://www.beebase.org/) | [9] |
| *N. vitripennis* | 1.0 | 1.0 | [http://www.hgsc.bcm.tmc.edu](http://www.hgsc.bcm.tmc.edu/) | [10] |
| *A. pisum* | 1.0 | 1.0 | http://www.hgsc.bcm.tmc.edu | - |
| *P. humanus humanus* | 1.0 | 1.2 | [http://vectorbase.org](http://vectorbase.org/) | [11] |
| *D. pulex* | 06.09.05 | 06.09.05 | [http://wfleabase.org](http://wfleabase.org/) | - |
| *C. elegans* | WS190.5.4 | WS190.5.4 | [http://www.ensembl.org](http://www.ensembl.org/) | [12] |
| *C. capitata* | 1.0 | 1.0 | [http://genome.jgi-psf.org](http://genome.jgi-psf.org/) | - |
| *L. gigantea* | 1.0 | 1.0 | [http://genome.jgi-psf.org](http://genome.jgi-psf.org/) | - |
| *A. californica* | 2.0 | - | [http://www.ncbi.nlm.nih.gov](http://www.ncbi.nlm.nih.gov/) | Broad Institute |
| *H. sapiens* | 36.54 | 36.54 | [http://www.ensembl.org](http://www.ensembl.org/) | [13] |
| *M. musculus* | 37.54 | 37.54 | [http://www.ensembl.org](http://www.ensembl.org/) | [14] |
| *D. rerio* | 8.54 | 8.54 | [http://www.ensembl.org](http://www.ensembl.org/) | - |
| *C. intestinalis* | 2.54 | 2.54 | [http://genome.jgi-psf.org](http://genome.jgi-psf.org/) | [15] |
| *S. purpuratus* | 2.1 | 2.1 | [http://www.hgsc.bcm.tmc.edu](http://www.hgsc.bcm.tmc.edu/) | [16] |
| *N. vectensis* | 1.0 | 1.0 | [http://genome.jgi-psf.org](http://genome.jgi-psf.org/) | [17] |
| *T. adhaerens* | 1.0 | 1.0 | [http://genome.jgi-psf.org](http://genome.jgi-psf.org/) | [18] |
| *A. queenslandica* | - | - | - | [19] |
| *M. brevicollis* | 1.0 | 1.0 | [http://genome.jgi-psf.org](http://genome.jgi-psf.org/) | [20] |
| *S. cerevisiae* | 1.01.54 | 1.01.54 | [http://www.ensembl.org](http://www.ensembl.org/) | [21] |
| *A. niger* | 1.0 | 1.0 | [http://genome.jgi-psf.org](http://genome.jgi-psf.org/) | - |
| *S. commune* | 1.0 | 1.0 | [http://genome.jgi-psf.org](http://genome.jgi-psf.org/) | - |
| *D. discoideum* | 1.0 | 1.0 | [http://dictybase.org](http://dictybase.org/) | [22] |
| *C. merolae* | 1.0 | 1.0 | <http://merolae.biol.s.u-tokyo.ac.jp/> | [23] |
| *P. tricornutum* | 2.0 | 2.0 | [http://genome.jgi-psf.org](http://genome.jgi-psf.org/) | [24] |
| *A. thaliana* | 9.0 | - | [http://arabidopsis.org](http://arabidopsis.org/) | [25] |

Prokaryotic genome sequences were downloaded from: ftp://ftp.ncbi.nlm.nih.gov/genomes/Bacteria/

1. Adams MD, Celniker SE, Holt RA, Evans CA, Gocayne JD, et al. (2000) The genome sequence of *Drosophila melanogaster*. Science 287: 2185-2195.

2. Clark AG, Eisen MB, Smith DR, Bergman CM, Oliver B, et al. (2007) Evolution of genes and genomes on the *Drosophila* phylogeny. Nature 450: 203-218.

3. Richards S, Liu Y, Bettencourt BR, Hradecky P, Letovsky S, et al. (2005) Comparative genome sequencing of *Drosophila pseudoobscura*: chromosomal, gene, and cis-element evolution. Genome Res 15: 1-18.

4. Nene V, Wortman JR, Lawson D, Haas B, Kodira C, et al. (2007) Genome sequence of *Aedes aegypti*, a major arbovirus vector. Science 316: 1718-1723.

5. Lawson D, Arensburger P, Atkinson P, Besansky NJ, Bruggner RV, et al. (2009) VectorBase: a data resource for invertebrate vector genomics. Nucleic Acids Res 37: D583-587.

6. Holt RA, Subramanian GM, Halpern A, Sutton GG, Charlab R, et al. (2002) The genome sequence of the malaria mosquito *Anopheles gambiae*. Science 298: 129-149.

7. Xia Q, Zhou Z, Lu C, Cheng D, Dai F, et al. (2004) A draft sequence for the genome of the domesticated silkworm (*Bombyx mor*i). Science 306: 1937-1940.

8. Richards S, Gibbs RA, Weinstock GM, Brown SJ, Denell R, et al. (2008) The genome of the model beetle and pest *Tribolium castaneum*. Nature 452: 949-955.

9. Honeybee Genome Sequencing Consortium. (2006) Insights into social insects from the genome of the honeybee *Apis mellifera*. Nature 443: 931-949.

10. Werren JH, Richards S, Desjardins CA, Niehuis O, Gadau J, et al. (2010) Functional and evolutionary insights from the genomes of three parasitoid *Nasonia* species. Science 327: 343-348.

11. Kirkness EF, Haas BJ, Sun W, Braig HR, Perotti MA, et al. (2010) Genome sequences of the human body louse and its primary endosymbiont provide insights into the permanent parasitic lifestyle. Proc Natl Acad Sci U S A.

12. *C. elegans* Sequencing Consortium. (1998) Genome sequence of the nematode *C. elegans*: a platform for investigating biology. Science 282: 2012-2018.

13. Venter JC, Adams MD, Myers EW, Li PW, Mural RJ, et al. (2001) The sequence of the human genome. Science 291: 1304-1351.

14. Waterston RH, Lindblad-Toh K, Birney E, Rogers J, Abril JF, et al. (2002) Initial sequencing and comparative analysis of the mouse genome. Nature 420: 520-562.

15. Dehal P, Satou Y, Campbell RK, Chapman J, Degnan B, et al. (2002) The draft genome of *Ciona intestinalis*: insights into chordate and vertebrate origins. Science 298: 2157-2167.

16. Sodergren E, Weinstock GM, Davidson EH, Cameron RA, Gibbs RA, et al. (2006) The genome of the sea urchin *Strongylocentrotus purpuratus*. Science 314: 941-952.

17. Putnam NH, Srivastava M, Hellsten U, Dirks B, Chapman J, et al. (2007) Sea anemone genome reveals ancestral eumetazoan gene repertoire and genomic organization. Science 317: 86-94.

18. Srivastava M, Begovic E, Chapman J, Putnam NH, Hellsten U, et al. (2008) The *Trichoplax* genome and the nature of placozoans. Nature 454: 955-960.

19. Srivastava M, Simakov O, Chapman J, Fahey B, Gauthier MEA, et al. (2010) The *Amphimedon queenslandica* genome and the evolution of animal complexity. Nature, *in press*.

20. King N, Westbrook MJ, Young SL, Kuo A, Abedin M, et al. (2008) The genome of the choanoflagellate *Monosiga brevicollis* and the origin of metazoans. Nature 451: 783-788.

21. Mewes HW, Albermann K, Bahr M, Frishman D, Gleissner A, et al. (1997) Overview of the yeast genome. Nature 387: 7-65.

22. Eichinger L, Pachebat JA, Glockner G, Rajandream MA, Sucgang R, et al. (2005) The genome of the social amoeba *Dictyostelium discoideum*. Nature 435: 43-57.

23. Matsuzaki M, Misumi O, Shin IT, Maruyama S, Takahara M, et al. (2004) Genome sequence of the ultrasmall unicellular red alga *Cyanidioschyzon merolae* 10D. Nature 428: 653-657.

24. Bowler C, Allen AE, Badger JH, Grimwood J, Jabbari K, et al. (2008) The *Phaeodactylum* genome reveals the evolutionary history of diatom genomes. Nature 456: 239-244.

25. Arabidopsis Genome Initiative. (2000) Analysis of the genome sequence of the flowering plant *Arabidopsis thaliana*. Nature 408: 796-815.
